# *Allium mongolicum* Regel Enhances Serum Immunity, Antioxidant, and Biochemical Indicators of Meat Sheep Achieved by Rumen Microbiota Regulation

**DOI:** 10.3390/ani15172491

**Published:** 2025-08-25

**Authors:** Xiaoyuan Wang, Chen Bai, Khas Erdene, Yankai Zheng, Qina Cao, Guoli Han, Changjin Ao

**Affiliations:** Key Laboratory of Animal Nutrition and Feed Science, College of Animal Science, Inner Mongolia Agricultural University, Zhaowuda Road 306, Saihan District, Hohhot 010018, China

**Keywords:** *Allium mongolicum* Regel, microbiota, rumen fluid transplantation, immunity and antioxidant capacity, meat sheep

## Abstract

Feeding *Allium mongolicum* Regel (**AMR**) could improve lamb growth, immunity, and antioxidant capacity. These effects were supposed to be mediated by the rumen microbiota, as reported in our previous studies, but further verification is required. The purpose of this study was to verify whether changes in serum immunity, antioxidant, and biochemical indicators of meat sheep mediated by AMR are achieved via rumen microbiota regulation. The experiment included two phases. In phase I, twelve 90-day-old male lambs (25 ± 1 kg) were used as rumen fluid donors and consumed a basal diet with 15 g/day AMR for 135 days to induce changes in their rumen microbiota. In phase II, thirty 90-day-old male lambs (23 ± 2 kg) were split into three groups (n = 10 each): the control group (**CON**) fed the basal diet; the AMR-supplemented group (**AMG**) fed the basal diet supplemented with 15 g/day of AMR; and the rumen fluid recipient group (**RTG**) fed the basal diet and received rumen fluid transplantation. The CON and AMG groups received four oral infusions of 250 mL saline, while the RTG group received four oral infusions of 250 mL donor rumen fluid. Phase II lasted for 75 days, and the blood samples were collected on the last day. Rumen fluid transplantation was performed every 15 days, with a total of four infusions of 250 mL each. The results showed that the final body weight and average daily gain (**ADG**) of the AMG and RTG groups were higher than those of the CON group (*p* < 0.05), while there were no significant differences between the AMG and RTG groups (*p* > 0.05). On day 30, the levels of interleukin-10 (**IL-10**), immunoglobulin A (**IgA**), immunoglobulin M (**IgM**), and immunoglobulin G (**IgG**) in the AMG and RTG groups were increased (*p* < 0.05), and malondialdehyde (**MDA**) was significantly decreased (*p* < 0.05), with no significant differences between the AMG and RTG groups. On day 60, total antioxidant capacity (**T-AOC**), IgM, IgG, and catalase (**CAT**) were increased in the AMG and RTG groups, while tumor necrosis factor alpha (**TNF-α**), low-density lipoprotein cholesterol (**LDL-C**), and blood urea nitrogen (**BUN**) were decreased (*p* < 0.05), and there were no significant differences between the AMG and RTG groups (*p* > 0.05). High-density lipoprotein cholesterol (**HDL-C**) in the RTG group was significantly lower than in the CON and AMG groups, while the AMG group had significantly higher HDL-C than the CON group (*p* < 0.05). Microbial analysis indicated that *Mitsuokella*, *VUNI01*, and *Caecibacter* were positively correlated with IgM; *Mitsuokella*, *CAG* 791, *Desulfovibrio R*, *Porcincola*, *VUNI01*, and *UBA* 7741 were negatively correlated with TNF-α; *CAG* 791 was positively correlated with T-AOC; *VUNI01* was positively correlated with CAT; MDA was negatively correlated with *Mitsuokella*; *Allisonella* and *UBA* 7741 were negatively correlated with HDL-C; and *Porcincola*, *VUNI01*, *Allisonella*, and *UBA* 7741 were negatively correlated with LDL-C. Therefore, the study indicates that both supplementation with AMR and transplantation of rumen fluid from sheep fed with AMR can enhance the immunity and antioxidant capacity of lambs by increasing the abundance of the aforementioned bacteria. It also verified that the improvement in immunity and antioxidant capacity mediated by *Allium mongolicum* Regel is driven by the rumen microbiota.

## Simple Summary

*Allium mongolicum* Regel, a typical herbaceous plant of the *Allium* genus, is rich in active compounds such as flavonoids, polyphenols, and organic acids. Over the past decade, it has been shown to enhance the growth performance and health status of meat sheep while regulating the rumen microbiota. However, it remains uncertain whether these improvements in growth and health mediated by *Allium mongolicum* Regel are primarily driven by the microbiota. Understanding the dynamic changes in key rumen microorganisms during alterations in immune response and antioxidant status in meat sheep is critical for the production practices of meat sheep. This study elucidates the effects of key microorganisms on serum immune, antioxidant, and biochemical indicators in meat sheep by transplanting rumen fluid from meat sheep fed *Allium mongolicum* Regel. The results indicate that certain key rumen microorganisms influence the growth, immune response, and antioxidant status of meat sheep. In the future, microbial preparations could be developed to enhance the practical production performance of meat sheep.

## 1. Introduction

Intensive farming methods have increased sheep meat production, but they have brought new health challenges, such as physiological stress in animals, welfare needs, and oxidative stress [1]. Antibiotics were once seen as a common strategy to address the drawbacks of intensive farming, but due to their tendency to induce microbial resistance, leading to environmental pollution and serious threats to human health, their large-scale use in livestock has been widely banned globally over the past two decades [2]. Probiotics, prebiotics, postbiotics, organic acids, and plant extracts, among others, are used as nutritional strategies to replace antibiotics due to their safety for human health or their ability to promote livestock growth and health [3,4,5,6]. Plant extracts are particularly notable because of their wide distribution and natural, renewable properties, meeting consumer expectations and aligning with sustainable development strategies. More importantly, plant extracts are rich in bioactive components, including polyphenols, flavonoids, and sterols [7]. These phytogenic substances exhibit inherent antioxidant properties that effectively enhance immune, antioxidant, and biochemical indicators in ruminants [8].

*Allium mongolicum* Regel (**AMR**), a typical medicinal and edible plant commonly found in deserts, gobi, and semi-arid high-altitude regions [9], is rich in protein, amino acids, flavonoids, and polyphenols [10,11]. Our previous study indicated that AMR could regulate lamb immunity, antioxidants, and meat flavor [12,13]. Muqier et al. [14] found that AMR can increase serum levels of interleukin-1β (**IL-1β**), interleukin-2 (**IL-2**), interleukin-6 (**IL-6**), and interferon gamma (**IFN-γ**) in sheep, thereby enhancing both non-specific and specific immunity to varying degrees. Additionally, supplementation with AMR can promote energy metabolism and thereby enhance animal growth performance by increasing the levels of growth hormone and insulin-like growth factor-1 in the serum of sheep, while also altering the rumen microbiota, such as *Tenericutes* [15,16]. Similarly, in an in vitro study, Wang et al. [17] found that AMR can reduce the abundance of hydrogenation bacteria such as *Butyrivibrio proteoclasticus*, *Butyrivibrio fibrisolvens*, *Ruminococcus albus*, and *Clostridium aminophilum*, while it enhanced the rumen fermentation profile. The rumen microbiota has the potential to regulate animal physiological characteristics. Although numerous studies have elucidated the positive effects of AMR supplementation on lamb growth, immunity, and antioxidation, as well as its regulatory effect on the rumen microbiota, it is unknown whether the changes in health status indicators were regulated by the rumen microbiota. Wang et al. [18] investigated the interplay between rumen microbes and the host’s immune and antioxidant status but could not identify the key microbiota driving these changes. Su et al. [19] reported that the addition of an herbal formula could increase the activities of glutathione peroxidase (**GSH-Px**) and catalase (**CAT**) while increasing the abundances of *Prevotella* and *Succiniclasticum*, but the relationship between microbiota and immune capacity was not clarified [19]. Based on this background, it is necessary to investigate the rumen microbiota’s regulatory effect on immunity and antioxidant capacity of meat sheep mediated by AMR supplementation.

Therefore, we hypothesize that *Allium mongolicum* Regel affects lamb immunity and antioxidation by regulating the rumen microbiota. The objective of this study is to verify, through rumen fluid transplantation, whether changes in the rumen microbiota mediated by *Allium mongolicum* Regel are the driving factors in enhancing lamb immunity, antioxidation, and biochemistry indicators.

## 2. Materials and Methods

### 2.1. Animal Ethics Statement

All procedures were approved by the Animal Ethics and Welfare Committee of Inner Mongolia Agricultural University (Guidelines No. NND2022049), adhering to the Chinese Ministry of Science and Technology’s regulations on experimental animal ethics.

### 2.2. Preparation of AMR Powder

The AMR was harvested from Bayanhot, Alxa Left Banyannuur, Inner Mongolia, China, during its peak phenological stage from July to September. The freshly harvested AMR was evenly distributed and dried in a 65 °C oven (DHG-9070, Shanghai Yiheng Instrument Group, Shanghai, China). This controlled thermal processing was optimized to maintain the structural integrity of bioactive flavonoids, thereby preserving their physicochemical stability and biological efficacy. The dried AMR was ground using an herbal grinder, model DFT-300 (Shanghai Xinnuo Instrument Group, Shanghai, China), and then passed through an 80-mesh sieve (Shaoxing Shangyu Instrument Co., Ltd., Shaoxing, China) to ensure particle homogeneity. The resulting AMR was stored in bags at 4 °C until use. The nutritional composition of AMR in Table 1 was obtained from Liu et al. [12].

### 2.3. Experimental Design and Animal Management

#### 2.3.1. Animal Rearing

All lambs were from the Commercial Meat Breeding Sheep Company (Bayannuur, Inner Mongolia, China). The entire experiment (phase I and phase II) was conducted over a period of 135 days. In trial phase I, twelve Small-tailed Han × Dorper (25 ± 1 kg, **BW**) males aged 90 days were selected as donor lambs. Lambs were fed a diet containing 15 g/d of AMR powder per lamb for 135 days. The dosage of 15 g/d of AMR additives was determined through previous animal experiments [16,21,22]. Upon reaching day 60 of trial phase I, phase II was launched, consisting of a 15-day adaptation stage and a subsequent 60-day rumen fluid transplantation phase. A total of thirty male lambs, aged 90 days and of the Small-tailed Han × Dorper lineage (23 ± 2 kg, BW), were chosen and randomly allocated to three distinct treatment groups, each consisting of ten lambs. The groups included three biological replicates, with each replicate consisting of 3, 3, and 4 lambs, respectively. The lambs in the first group were provided the basal diet and orally infused with rumen fluid from donor lambs to investigate the effects of microbial changes on immunity and antioxidant capacity through rumen fluid transplantation (RTG, n = 10); the lambs in the second group were provided the basal diet supplemented with 15 g/day per lamb of AMR powder and orally infused with saline, used for comparison with the RTG group to verify the effects of microbial changes on immunity and antioxidant capacity (AMG, n = 10); and the lambs in the third were provided the basal diet and orally infused with saline, serving as a control for comparison with the AMG and RTG groups without altering the rumen microbiota (CON, n = 10). Infusion and AMR feeding treatments were conducted on the 16th day in phase II. The infusion interval was 15 days, with each infusion involving 250 mL of saline or rumen fluid. Infusion was performed 4 times, with a total infused volume of 1 L. Each lamb in the RTG group received a cumulative total of 1 L of rumen fluid, and each lamb in the CON and AMG groups received a cumulative total of 1 L of saline.

**Figure 1:**
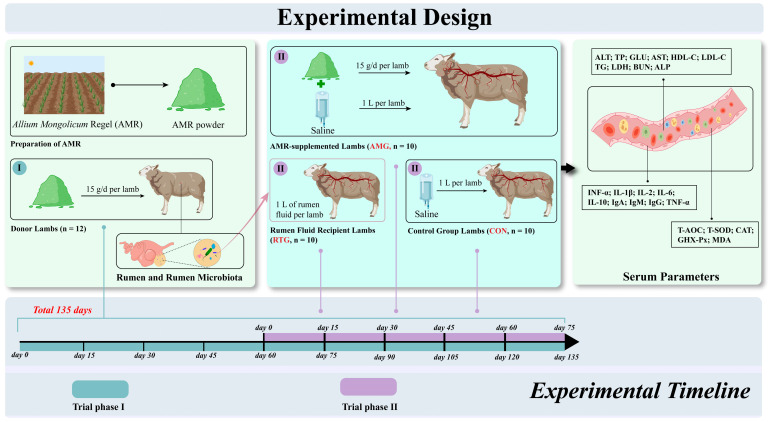
Experimental design. From left to right, the picture shows the establishment of the ruminal fluid donor model, the treatment methods of each group, and the serum-related measurement parameters. The different colors on the timeline represent various phases of the experiment. During trial phase I, which lasted 135 days, a rumen fluid transplantation donor model was established. On day 60 of trial phase I, a 15-day adaptation period for trial phase II began. Subsequently, in trial phase II, rumen fluid transplantations were conducted every 15 days, totaling five transplantations, each involving 200 mL of rumen fluid or saline.

Each replicate house measured 3 m × 4 m to ensure sufficient space for movement and animal welfare. All lambs had free access to water and were housed at the sunny end of the barn to ensure at least 10 h per day of light exposure. Additionally, the house’s temperature was controlled at 20 °C ± 5%, and humidity was maintained at 50% ± 10% to minimize environmental interference. The detailed experimental design is illustrated in Figure 1, and the basal diet’s detailed formulation and nutrient profile are presented in Table 2.

#### 2.3.2. Transplantation Procedure

On the day of transplantation, all lambs underwent fasting before each procedure. Rumen fluid and saline were transplanted using a ruminal cannula (MDW16, Chengdu Huazhi Kaiwu Technology Co., Ltd., Chengdu, China) based on the methodology presented by Liu et al. [23]. Rumen fluid was collected from twelve donor lambs and immediately combined with CO_2_ in a flask. For each donor, 350 mL of rumen fluid was aspirated, discarding the initial 100 mL to avoid contamination. Recipients in the RTG group received 250 mL of rumen fluid per transplantation, while the CON and AMG groups received 250 mL of saline.

### 2.4. Assessment of Growth Performance

During the experimental period, feed offered and refusals were recorded, and lamb body weights were measured at 15-day intervals. The feed conversion ratio was determined from the weight gain of the lambs. The DM content was assessed using Method No. 930.15 outlined by the Association of Official Analytical Chemists (AOAC, 2005) [24]. The DM content of the fresh feed sample was calculated using the following model:

$$\mathrm{DM}=\frac{\mathrm{Dry}\mathrm{weight}}{\mathrm{Initial}\mathrm{weight}}\times 100\%$$

The feed intake was calculated using the following model:

Feedintake=Feedoffered−Feedrefusals

The DM intake (DMI) was calculated using the following model:

$$\mathrm{DMI}=\left(\mathrm{Feed}\mathrm{intake}\times \mathrm{DM}\right)$$

### 2.5. Chemical Analysis of Feed Samples

The **CP** content in the feed samples was determined by multiplying the nitrogen content by 6.25. The levels of Ca, P, NDF, ADF, and DM in the feed samples were quantified using the methods outlined by the Association of Official Analytical Chemists (AOAC, 2005) [24].

### 2.6. Sample Acquisition and Processing

Blood samples were obtained from the lambs every 15 days using a non-anticoagulant blood collection tube with a 5 mL capacity (Huabo Medical Instruments Co., Ltd., Nanjing, China). After allowing the blood to stand for 30 min to separate the serum, it was centrifuged at 3000 r/min for 15 min and stored at −20 °C until analysis.

At the end of the experiment, all lambs were transported to a slaughterhouse. The rumen fluid was collected and subsequently filtered through four layers of gauze before being stored at −80 °C until DNA extraction.

### 2.7. Serum Indicator Measurements

Using antioxidant assay kits (Nanjing jiancheng Bioengineering Institute in Nanjing, China), the serum activities of total superoxide dismutase (**T-SOD**), GSH-Px, CAT, and total antioxidant capacity (**T-AOC**), along with malondialdehyde (**MDA**) levels, were evaluated in accordance with the manufacturer’s protocol. Using ELISA kits (Nanjing Jiancheng Bioengineering Institute in Nanjing, China), the levels of immunoglobulin A (**IgA**), immunoglobulin G (**IgG**), immunoglobulin M (**IgM**), IL-1β, IL-2, IL-6, interleukin-10 (**IL-10**), tumor necrosis factor alpha (**TNF-α**), and IFN-γ in serum were evaluated in accordance with the manufacturer’s protocol. Using commercial kits (Nanjing Jiancheng Bioengineering Institute in Nanjing, China), the levels of total protein (**TP**), ALB, glucose (**GLU**), blood urea nitrogen (**BUN**), triglycerides (**TG**), high-density lipoprotein cholesterol (**HDL-C**), low-density lipoprotein cholesterol (**LDL-C**), alanine aminotransferase (**ALT**), aspartate aminotransferase (**AST**), lactate dehydrogenase (**LDH**), and alkaline phosphatase (**ALP**) in serum were evaluated in accordance with the manufacturer’s protocol. The analyses were performed using a Mindray BS-480 fully automatic biochemical analyzer (Mindray Bio-Medical Electronics Co., Ltd., Shenzhen, China).

### 2.8. Microbial DNA Extraction and 16S rRNA Amplicon Sequencing

Using the OMEGA Soil DNA Kit (M5635-02) sourced from Omega Bio-Tek (Norcross, GA, USA), microbial DNA was extracted in accordance with the manufacturer’s protocol. The 16S rRNA V3-V4 region was amplified using the primers 338F (5′-barcode+ACTCCTACGGGAGGCAGCA-3′) and 806R (5′-GGACTACHVGGGTWTCTAAT-3′). The initial step of the PCR involved heating the template DNA to 98 °C for 5 min to guarantee its full denaturation. The amplification process was conducted over 25 cycles, each involving a denaturation step at 98 °C for 30 s, an annealing step at 52 °C for 30 s, and an extension step at 72 °C for 45 s. The analysis of PCR products was carried out using 2% agarose gel electrophoresis. The sequencing library was prepared using the TruSeq DNA LT Kit (Illumina, San Diego, CA, USA) without additional PCR. Amplicon libraries were sequenced using the Illumina NovaSeq PE250 platform. A 1 μL aliquot of the library was assessed for quality using an Agilent Bioanalyzer with the Agilent High Sensitivity DNA Kit (Agilent, Santa Clara, CA, USA). A qualified library should display a single peak devoid of adapter contamination. Using the Quant-iT PicoGreen dsDNA Assay Kit (Thermo Fisher Scientific, Waltham, MA, USA), library quantification was carried out on a Promega QuantiFluor instrument (Madison, WI, USA), with the goal of achieving a concentration greater than 2 nM. Paired-end sequencing of qualified libraries was conducted on the Illumina NovaSeq platform with read lengths of 2 × 250 bp, using the NovaSeq 6000 SP Reagent Kit (500 cycles).

### 2.9. Data Analysis

All data were previously subjected to the Shapiro–Wilk test for normality, and all conformed to a normal distribution. Data on growth performance and serum indicators were analyzed using one-way analysis of variance (**ANOVA**) in SAS 9.21 software (Institute Inc., Cary, NC, USA). Duncan’s multiple range test was employed for multiple comparisons. Results are presented as mean and standard error, with differences considered statistically significant at *p* < 0.05. The statistical analysis utilized the linear model as outlined by Hu et al. [25]:

$$Y_{ij}=\mu +Ti+ɛij$$

where *Yij* represents the dependent variable; *μ* represents the overall mean (n = 6); *Ti* represents the fixed effect of dietary treatment; and *ɛij* is the residual error.

The microbial diversity data and difference visualization are analyzed on a professional platform (https://www.genescloud.cn/home).

## 3. Results

### 3.1. Growth Performance

The impact of AMR supplementation or rumen fluid transplantation on lamb growth performance is referenced from an unpublished manuscript (Wang et al., Inner Mongolia Agricultural University, China) and summarized in Table 3. Compared to the CON group, the final body weights in the AMG and RTG groups were significantly higher (*p* < 0.05), and there was no significant difference between the AMG and RTG groups. Additionally, the average daily gain (**ADG**) of the AMG and RTG groups increased by 23.53% and 32.35%, respectively, compared to the CON group (*p* < 0.05), and there was no significant difference between the AMG and RTG groups.

### 3.2. Serum Immune Indicators

Table 4 shows that at 0 days, there were no significant differences in immune indicators between the groups (*p* > 0.05). At 30 days, compared to the CON group, the AMG and RTG groups had significantly higher levels of IL-10, IgA, IgM, and IgG (*p* < 0.05), with no significant differences between the AMG and RTG groups (*p* > 0.05). At 60 days, compared to the CON group, the AMG and RTG groups had significantly lower levels of TNF-α (*p* < 0.01) and significantly higher levels of IgA, IgM, and IgG (*p* < 0.01). Similarly, there were no significant differences in TNF-α, IgA, IgM, and IgG levels between the AMG and RTG groups (*p* > 0.05).

### 3.3. Serum Antioxidant Indicators

Table 5 presents the serum antioxidant levels on days 0, 30, and 60. On day 0, there were no significant differences in antioxidant indicators among the groups (*p* > 0.05). On day 30, compared to the CON group, the MDA levels in the AMG and RTG groups were significantly lower (*p* < 0.001), with no significant difference between the AMG and RTG groups (*p* > 0.05). By day 60, compared to the CON group, the T-AOC and CAT levels in the AMG and RTG groups were significantly higher (*p* < 0.001), and the MDA levels were significantly lower (*p* < 0.001). There were no significant differences between the AMG and RTG groups (*p* > 0.05).

### 3.4. Serum Biochemical Indicators

Table 6 shows the levels of serum biochemical indicators on day 0, day 30, and day 60. On day 0, there were no significant differences in biochemical indicators among the groups (*p* > 0.05). On day 30, the serum antioxidant levels in the CON group were not significantly different from those in the AMG group or the RTG group (*p* > 0.05). By day 60, the HDL-C levels in the AMG group were significantly higher than those in the CON group (*p* < 0.05), while the RTG group had significantly lower levels than both the CON group and the AMG group (*p* < 0.05). The level of LDL-C in both the AMG group and the RTG group showed significant reductions (*p* < 0.05), with no significant difference between the two groups (*p* > 0.05). For the BUN indicator, both the AMG group and the RTG group showed significant reductions compared to the CON group (*p* < 0.001), and there was no significant difference between the AMG group and the RTG group (*p* > 0.05).

### 3.5. Venn Map

**Figure 2:**
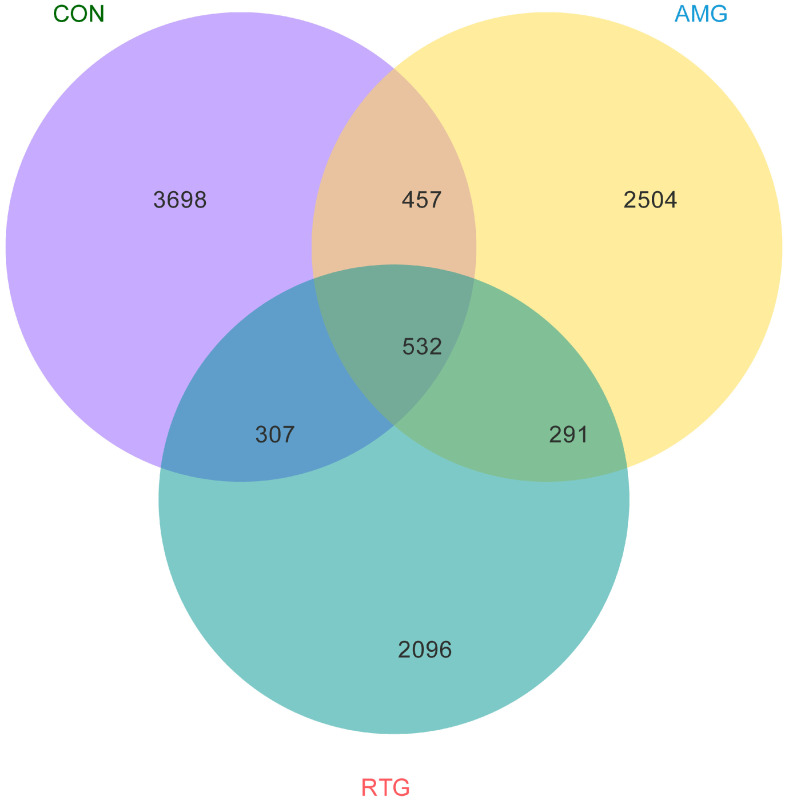
Differences in the distribution of Operational Taxonomic Units (OTUs) among groups, displayed using a Venn diagram. Each colored block represents a specific group, with overlapping areas between blocks indicating shared OTUs between the corresponding groups. Numbers within each block denote the total quantity of OTUs in that block, including those unique and shared. CON = Infusion with saline only; AMG = infusion with saline and supplementation with *Allium mongolicum* Regel; RTG = infusion with rumen fluid from donor sheep fed *Allium mongolicum* Regel.

Operational Taxonomic Unit (**OTU**) distribution patterns across the three experimental groups are shown in Figure 2. The CON group exhibited the highest OTU richness, with 3698 observed units, followed by the AMG group (2504 OTUs) and RTG group (2096 OTUs). The Venn diagram revealed substantial overlaps in the microbial community: 457 OTUs were shared exclusively between the CON and AMG groups, 307 between the CON and RTG groups, and 291 between the AMG and RTG groups. A core set of 532 OTUs was conserved across all three groups, representing 14.4%, 21.3%, and 25.4% of the total OTUs in the CON, AMG, and RTG groups, respectively.

### 3.6. Microbial Diversity Analysis

#### 3.6.1. Multi-Sample Species Classification

**Figure 3:**
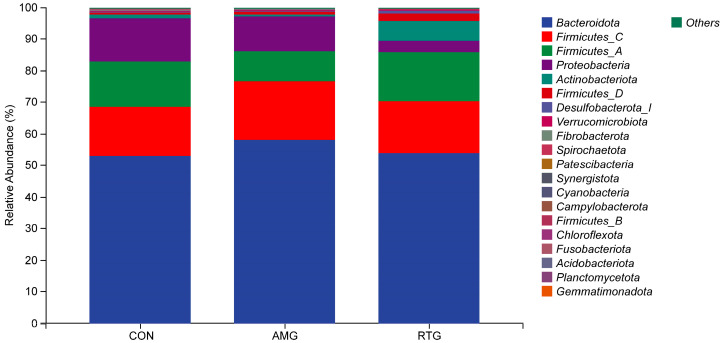
Relative abundance of the top 20 bacterial phyla in the rumen. The *x*-axis displays the various experimental groups, whereas the *y*-axis indicates the proportional presence of bacterial phyla. Each bar corresponds to one of the top 20 abundant bacterial taxa in the rumen. CON = Infusion with saline only; AMG = infusion with saline and supplementation with *Allium mongolicum* Regel; RTG = infusion with rumen fluid from donor sheep fed *Allium mongolicum* Regel.

Microbial diversity analysis revealed the TOP 20 ruminal dominant bacteria phyla (Figure 3). The combined abundance of *Bacteroidota*, *Firmicutes C*, *Fimicutes A*, *Proteobacteriota*, *Actinobacteriota*, *Fimicutes D*, *Desulfobacterota I*, *Verrucomicrobiota*, *Fibrobacterota*, *Spirochaetota*, *Patescibacteria*, *Synergistota*, *Cyanobacteria*, *Campylobacterota*, *Fimicutes B*, *Chloroflexota*, *Fusobacteriota*, *Acidobacterioda*, *Planctomycetota*, and *Gemmatimonadota* accounted for over 90% of the microbial community. A significant increase in the relative abundance of *Firmicutes D* was observed in the RTG group compared to the CON group (*p* < 0.05).

**Figure 4:**
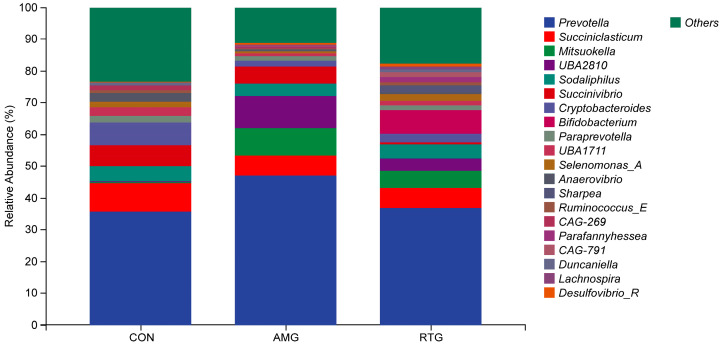
Relative abundance of the top 20 bacterial genera in the rumen. The *x*-axis displays the various experimental groups, whereas the *y*-axis indicates the proportional presence of bacterial genera. Each bar corresponds to 1 of the top 20 abundant bacterial taxa in the rumen. CON = Infusion with saline only; AMG = infusion with saline and supplementation with *Allium mongolicum* Regel; RTG = infusion with rumen fluid from donor sheep fed *Allium mongolicum* Regel.

Microbial profiling identified the top 20 predominant ruminal bacterial genera (Figure 4), collectively representing over 90% of the microbiota. The dominant taxa included *Prevotella*, *Succiniclasticum*, *Mitsuokella*, *UBA* 2810, *Sodaliphilus*, *Succinivibrio*, *Cryptobacteroides*, *Bifidobacterium*, *Paraprevotella*, *UBA* 1711, *Selenomonas A*, *Anaerovibrio*, *Sharpea*, *Ruminococcus E*, *CAG* 791, *Duncaniella*, *Lachnospira*, and *Desulfovibrio R*. The abundance of the genus *Succiniclasticum* was significantly lower in both the AMG and RTG groups compared with the CON group (*p* < 0.05). The AMG group exhibited higher abundances of *Mitsuokella*, *Sodaliphilus*, and *UBA2810* relative to the CON group (*p* < 0.05), whereas the RTG group showed significant enrichment of *Parafannyhessea*, *CAG* 791, *Duncaniella*, and *Lachnospira* relative to the CON group (*p* < 0.05).

#### 3.6.2. Alpha Diversity

**Figure 5:**
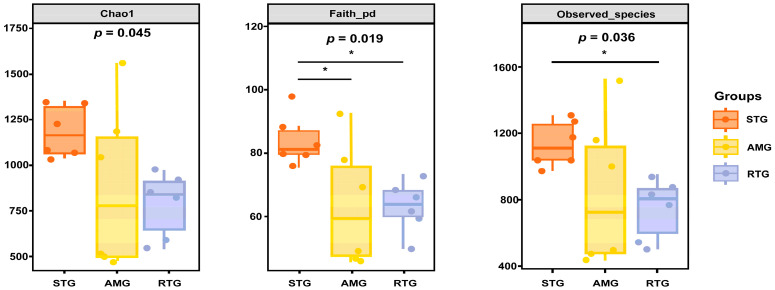
Alpha diversity. Each panel represents a specific alpha-diversity index, labeled in the gray area at the top. Below each index label, the numbers indicate the Kruskal–Wallis test *p*-values. Pairwise group comparisons are marked with significant results from Dunn’s test post hoc analysis, as indicated by specific markers. CON = Infusion with saline only; AMG = infusion with saline and supplementation with *Allium mongolicum* Regel; RTG = infusion with rumen fluid from donor sheep fed *Allium mongolicum* Regel; 0.01 < *p* < 0.05 and *p* < 0.01 are denoted by * and **, respectively.

Alpha diversity is a comprehensive metric for assessing species richness and evenness. This metric is derived from the OTUs generated from each sample, which involves the random selection of sample sequences and the construction of curves based on sequence counts and the corresponding OTUs. Notably, the higher Chao1 index, Faith’s phylogenetic diversity (Faith_pd), and observed species count in the CON group indicate that it has a higher microbial diversity and richness compared to the AMG and RTG groups (Figure 5). These findings suggest that the CON group exhibits higher species richness and diversity.

#### 3.6.3. Beta Diversity

**Figure 6:**
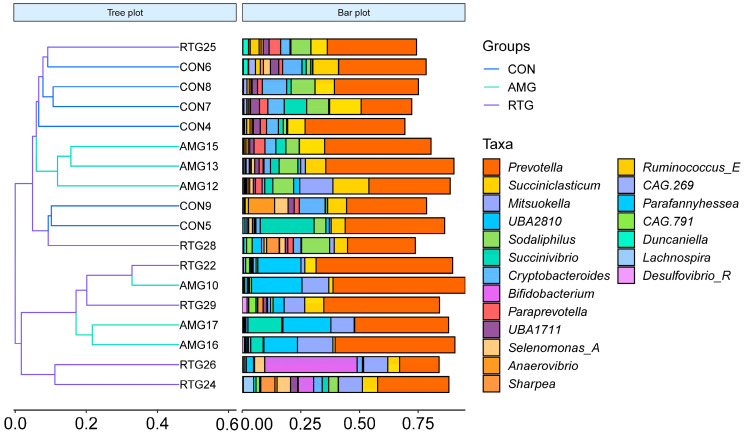
Hierarchical clustering analysis of the top 20 genera. The samples are clustered based on their similarities, with shorter branch lengths indicating greater similarity. The right panel displays a stacked bar chart of the top 20 genera, ranked by abundance. CON = Infusion with saline only; AMG = infusion with saline and supplementation with *Allium mongolicum* Regel; RTG = infusion with rumen fluid from donor sheep fed *Allium mongolicum* Regel.

A cluster heat map analysis was performed to determine the microbiota variations among the various groups, as illustrated in Figure 6. In the CON group, the increased abundance of *Cryptobacteroides* and the decreased abundance of *Mitsuokella* and *UBA2810* may have contributed to the formation of an autonomous cluster within the dendrogram. The abundance of *Bifidobacterium* was markedly higher in the RTG group than in both the CON and AMG groups.

#### 3.6.4. Linear Discriminant Analysis Effect Size (LEfSe) Analysis

**Figure 7:**
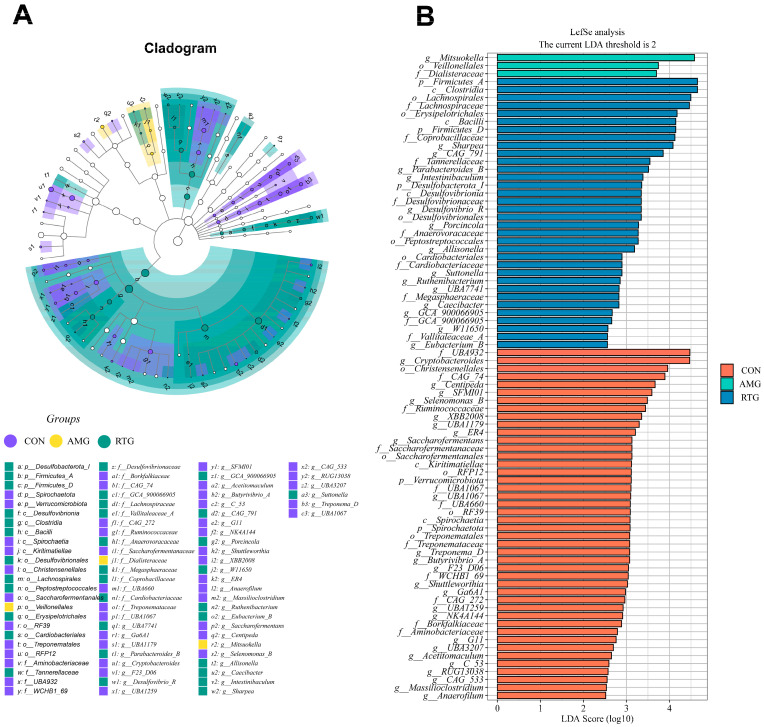
Linear discriminant analysis effect size (LEfSe) comparison of rumen microbiota. A cladogram illustrating taxonomic relationships derived from LEfSe analysis of 16s rRNA sequences (**A**). Distribution of the linear discriminant analysis results from the 18 samples in the CON, AMG, and RTG groups (**B**). CON = Infusion with saline only; AMG = infusion with saline and supplementation with *Allium mongolicum* Regel; RTG = infusion with rumen fluid from donor sheep fed *Allium mongolicum* Regel.

A LEfSe analysis was conducted to evaluate how species abundance influences diversity, identifying taxa that differ markedly in abundance between sample groups. The results are presented in Figure 7A,B. The analysis revealed 81 taxa with LDA scores greater than 2 across the three groups. In the AMG group, three taxa were identified, including *Mitsuokella*, *Veillonellales*, and *Dialisteraceae*. The identification process in the RTG group yielded 34 taxa, including *Firmicutes A*, *Clostridia*, *Lachnospiraceae*, *Bacilli*, *Firmicutes D*, *Coprobacillaceae*, *sharpea*, *CAG* 791, and *Tannerellaceae*. In the CON group, 44 taxa were identified, including *UBA* 932, *Cryptobacteroides*, *Christensenellales*, *CAG* 74, *Centipeda*, *SFM* 101, *Selenomonas B*, *Ruminococcaceae*, and *XBB* 2008.

### 3.7. Correlation Analysis Between Ruminal Bacteria and Serum Biochemical Indicators

**Figure 8:**
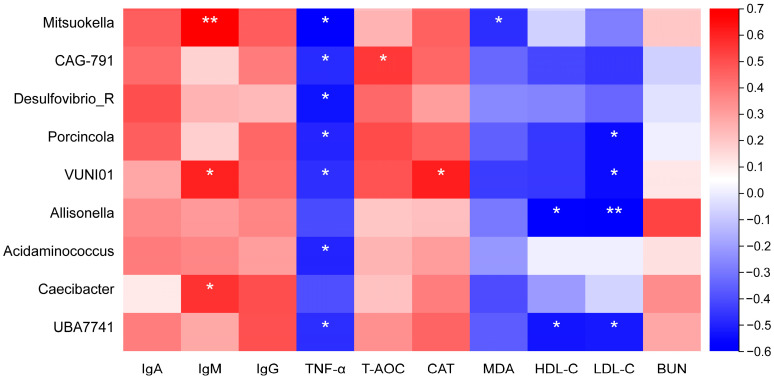
Correlation analysis between bacterial genera and serum indicators. The figure presents the Pearson correlation coefficients between bacterial genera (columns) and serum indicators (rows). Red represents positive correlations, while blue represents negative correlations. The depth of the color reflects the magnitude of the correlation, with darker colors representing stronger correlations. Statistically significant correlations are marked with asterisks (0.01 < *p* < 0.05 and *p* < 0.01 are denoted by * and **, respectively).

To investigate the functional correlation between bacteria and serum indices, a Pearson correlation matrix was created by calculating the correlations between bacteria exhibiting significant differences and serum immune, antioxidant, and biochemical indicators on day 60 (Figure 8). The results indicated a clear correlation between microbial structure and serum biochemical indicators (|*R*| > 0.4, *p* < 0.05). IgM was positively associated with *Mitsuokella* (*R* = 0.62, *p* < 0.01), *VUNI01* (*R* = 0.53, *p* < 0.05), and *Caecibacter* (*R* = 0.49, *p* < 0.05). Conversely, TNF-α showed a negative association with *Mitsuokella* (*R* = −0.59, *p* < 0.05), *CAG* 791 (*R* = −0.49, *p* < 0.05), *Desulfovibrio R* (*R* = −0.54, *p* < 0.05), *Porcincola* (*R* = −0.50, *p* < 0.05), *VUNI01* (*R* = −0.48, *p* < 0.05), and *UBA* 7741 (*R* = −0.48, *p* < 0.05). T-AOC was positively correlated with *CAG* 791 (*R* = 0.48, *p* < 0.05). CAT showed a positive association with *VUNI01* (*R* = 0.54, *p* < 0.05). MDA was negatively associated with *Mitsuokella* (*R* = −0.48, *p* < 0.05). HDL-C exhibited a negative association with *Allisonella* (*R* = −0.54, *p* < 0.05) and *UBA* 7741 (*R* = −0.53, *p* < 0.05), and LDL-C was negatively correlated with *Porcincola* (*R* = −0.55, *p* < 0.05), *VUNI01* (*R* = −0.55, *p* < 0.05), *Allisonella* (*R* = −0.60, *p* < 0.01), and *UBA7741* (*R* = −0.52, *p* < 0.05).

## 4. Discussion

The RTG and AMG groups exhibited significantly greater final body weight and ADG compared to the CON group. In fact, alterations in the microbiota communities mediated by active substances in AMR are beneficial for breaking down complex plant fibers and other nutrients, thereby improving the absorption efficiency of nutrients in ruminants [26]. The high protein level in AMR promotes the synthesis of microbial proteins by providing additional substrate for rumen protein-degrading bacteria [27,28] or by upregulating energy and amino acid metabolism pathways in rumen microbes, which increases the abundance of NADH-oxidant-related enzymes, thereby enhancing the efficiency with which microbes synthesize proteins [29]. Furthermore, high-protein AMR not only provides amino acids but may also lead to enrichment of amino-acid-metabolizing bacteria such as *Acidaminococcus* in the rumen or intestine, thereby improving the animal’s growth performance [30]. In the RTG group, the genus *Firmicutes D* showed higher abundance. The decomposition of cellulose and other complex carbohydrates relies heavily on *Firmicutes D* [31,32]. This enhancement may improve the host’s ability to utilize nutrients, subsequently promoting an increase in ADG [33]. Additionally, based on research by Redoy et al. [34] on garlic leaves, the antimicrobial properties of *Allium* plants reduce the utilization of nutrients by protozoa and harmful microorganisms in the rumen, thereby improving animal growth performance. In particular, polyphenols in AMR can inhibit rumen hydrogenation, reducing unnecessary energy waste [35].

The cellulolytic bacterium UBA 2810 enhances ruminant nutrition by efficiently degrading cellulose and plant polysaccharides, improving feed conversion efficiency, as shown by Chai et al. [36]. Their findings revealed a positive association between ruminal *UBA* 2810 abundance and ADG, suggesting its pivotal role in growth promotion through enhanced nutrient utilization. Our experimental results align with these observations, demonstrating significantly higher *UBA* 2810 colonization in the AMG group. This microbial modulation parallels the findings of Du et al. [16], who documented similar rumen microbial restructuring and subsequent ADG improvements in lambs supplemented with AMR and its extracts. Zhou et al. [37] confirmed that these effects may stem from microbial-induced enhancement of cellulose fermentation efficiency. More importantly, plant polyphenols promote beneficial microbiota in the rumen [38] and regulate the host’s energy metabolism pathways. Additionally, plant polyphenols can enhance short-chain volatile fatty acid production, which supports intestinal villus growth, boosts tight junction protein expression, and alleviates intestinal inflammation [39,40]. Collectively, dietary interventions can effectively manipulate rumen microbiota composition to potentiate the digestive utilization of fibrous substrates, ultimately translating to measurable improvements in livestock growth performance.

Previous studies indicate that rumen microbiota modulation exerts effects on host serum immune responses, antioxidant capacity [41], and biochemical stability [42]. Based on findings that AMR supplementation significantly alters rumen microbial composition [43], enhances immunity, and improves antioxidant activity in lambs, our study revealed that both the RTG and AMR groups exhibited higher serum IgM levels. Additionally, we observed a significant enrichment of the bacterial genus *Mitsuokella* in the RTG and AMG groups relative to the CON group. The genus *Mitsuokella* is recognized as a group of bacteria capable of producing short-chain fatty acids, particularly acetic acid, propionic acid, and butyric acid. Research indicates that these short-chain fatty acids can promote IgM secretion through indirect effects on B-cell differentiation and function, as well as by enhancing the production of plasma cells. Additionally, they may further stimulate T-cell-independent IgM secretion by increasing the activity of marginal zone B cells [44,45,46]. The proliferation of *Mitsuokella* may contribute to enhanced rumen microbial diversity, which is a critical determinant of host immune function [47]. Correlation analysis between microorganisms and immune indicators shows that the abundance of *Mitsuokella* is positively correlated with serum IgM levels, indicating that *Mitsuokella* plays a potentially positive role in enhancing host immunity.

Recent studies indicate a negative relationship between *Mitsuokella* abundance and MDA concentrations [48]. It can be surmised that this correlation arises from the recognized link between elevated MDA levels and cellular damage caused by oxidative stress [49]. The observed suppression of *Mitsuokella* proliferation under high MDA levels suggests that oxidative stress may impair both bacterial metabolic functions and host immune regulation through this microbial pathway. Conversely, enhanced *Mitsuokella* appears to confer protective effects by reducing MDA concentrations, potentially through antioxidant mechanisms. Our experimental findings revealed that both rumen fluid transplantation and dietary supplementation with AMR significantly decreased serum MDA levels in lambs. This dual approach suggests that AMR supplementation and RT may alleviate oxidative stress in lambs. The MDA reduction patterns align with previous observations of *Mitsuokella*-mediated antioxidant effects.

On day 60, lambs in the RTG and AMG groups exhibited significantly reduced serum TNF-α levels, and the genus *Acidaminococcus* showed higher abundance. The reduction is likely due to two primary factors highlighted in recent studies. Mao et al. [50] revealed a significant negative correlation between ruminal *Acidaminococcus* abundance and TNF-α, suggesting that an increase in *Acidaminococcus* may lead to TNF-α inhibition. The negative correlation between *Acidaminococcus*, *Mitsuokella*, *CAG* 791, and TNF-α indicates that there may be nutrient competition or production of anti-inflammatory substances to inhibit TNF-α secretion, which also reveals the regulatory effect of microbiota on pro-inflammatory factors in this study. Additionally, sulfur-containing bioactive compounds present in *Allium* species have been shown to exhibit strong TNF-α-inhibitory properties by regulating the NF-κB pathway [51]. Sánchez-Gloria et al. [52] have shown that supplementation with allicin at a dosage of 200 mg/kg (BW basis) reduced TNF-α levels while enhancing antioxidant capacity in male Wistar rats. Recent studies in ruminants confirm these findings, which demonstrated that garlic powder decreased serum TNF-α levels and improved immunity [53].

This study shows that the genus *CAG* 791 and T-AOC are significantly positively correlated in confined lambs from the RTG and AMG groups, suggesting that increasing *CAG* 791 may enhance antioxidant capacity by promoting the metabolic transformation of feed nutrients. Our result revealed that CAT activity at 60 days of age was significantly higher in the RTG and AMG groups compared to the CON group. The regulatory role of polyphenols and flavonoids in AMR in lambs’ antioxidant properties may account for this difference [7]. A study indicates that flavonoids and allicin in garlic and its extracts specifically eliminate hydroxyl radicals via sulfhydryl exchange mechanisms [54], which may directly account for the elevated serum CAT activity in lambs. Recent studies further confirm that AMR and its extracts can effectively inhibit H_2_O_2_-induced erythrocyte oxidative damage by enhancing serum CAT activity [22]. These findings reveal the close relationship between the antioxidant properties of *Allium* plants and ruminant physiological functions. Potential mechanisms may involve both the direct action of intestinally absorbed polyphenols and flavonoids that influence the immune system and the indirect enhancement of the host’s antioxidant defenses through modulation of rumen microbial community structure [55].

The HDL-C and LDL-C levels serve as biomarkers for cardiovascular risk assessment [56]. We found that, in the RTG and AMG groups, serum HDL-C levels were higher and LDL-C concentrations were lower than those in the CON group. This metabolic shift may be mediated by microbial modulation, as evidenced by the increased abundance of *UBA* 7714 and *Allisonella* following AMR supplementation and rumen transplantation, which mediated structural alterations in the microbial community. These microbial modifications potentially influenced metabolic functions, particularly lipid metabolism, thereby promoting HDL-C synthesis and LDL-C clearance. The observed HDL-C elevation might also be associated with AMR’s antioxidant properties. Antioxidant compounds have been shown to enhance HDL-C functionality by promoting reverse cholesterol transport [57]. Similarly, according to Muqier et al. [15], the AMR-derived flavonoids significantly reduced LDL-C levels in lambs, aligning with previous findings that these polyphenolic antioxidants modulate cholesterol metabolism [58]. The correlation between these microbes and lipoproteins indicates that they may affect metabolic functions, particularly lipid metabolism, thereby promoting the synthesis of HDL-C and the clearance of LDL-C. They may metabolize glucosinolates in AMR to produce volatile sulfur compounds, which can activate the expression of ATP-binding cassette transporter A1 (**ABCA1**) in hepatocytes—ABCA1 is a core protein for HDL synthesis, and its activation can promote the transfer of cholesterol from macrophages to HDL, enhancing the “reverse cholesterol transport” function of HDL [59,60,61,62]. In addition, AMR’s volatile sulfur compounds, metabolized by the microbiota, may play a role by interfering with bacterial membrane integrity or directly scavenging reactive oxygen species [63]. Meanwhile, flavonoids in AMR that are not fully metabolized exert antibacterial activity in recipient meat sheep through rumen fluid transplantation or inhibit the growth of harmful bacteria through hydroxyl substitution mode [64]. They can also remove free radicals via electron or hydrogen atom transfer [65,66]. These findings contribute to a better understanding of how AMR directly or indirectly affects immune, antioxidant, and biochemical indicators in sheep.

## 5. Conclusions

Rumen fluid transplantation from *Allium mongolicum* Regel-supplemented donors to recipients mediated microbial community restructuring and immunological regulation, closely mirroring the effects observed in directly supplemented lambs. These findings suggest that specific microbes in the rumen are crucial for inducing changes in host immune, antioxidant, and biochemical indicators. Consequently, microbial regulation emerges as an effective strategy to enhance ruminant health and productivity.

## Figures and Tables

**Table 1:** Nutritional composition of *Allium mongolicum* Regel (% DM basis) [20].

Item	Content
CP	32.55
Ether extract	6.25
NDF	17.55
ADF	15.87
Ca	0.97
P	0.58

**Table 2:** Composition and nutrient levels of the basal diet (DM basis).

Item	Content
Ingredients (%)	
Corn silage	19.67
Alfalfa meal	16.35
Corn stalk	10.01
Wheat bran	7.30
Corn	25.20
Soybean meal	14.53
Extruded soybeans	1.83
Premixes ^1^	2.48
NaCl	1.50
Limestone	1.13
Total	100.00
Nutrients levels	
Metabolic energy (MJ/kg)	11.82
CP (%)	14.50
Ether extract (%)	2.50
NDF (%)	34.60
ADF (%)	16.43
OM (%)	15.00
Calcium (%)	2.00
Phosphorus (%)	0.80

^1^ Premixes = Nutritional composition of premix per kilogram: 30.00 mg of Mn (as manganese), 25.00 mg of Fe (as ferrous sulfate), 29.00 mg of Zn (as zinc sulfate), 8.00 mg of Cu (as copper sulfate), 0.10 mg of Co (as cobalt sulfate), 0.45 mg of I (as potassium iodide), 3200 IU of Vitamin A, 1200 IU of Vitamin D, 20 IU of Vitamin E.

**Table 3:** Effects of supplementation with *Allium mongolicum* Regel or rumen fluid transplantation on growth performance of lambs.

Item ^1^	Treatments ^2^			SEM	*p*-Value
	CON	AMG	RTG		
Initial body weight, kg	22.47	22.53	24.18	0.65	0.128
Final body weight, kg	44.78 ^b^	48.33 ^a^	49.87 ^a^	0.69	0.005
Dry matter intake, kg/d	1.61	1.56	1.58	0.05	0.702
ADG, kg/d	0.34 ^b^	0.42 ^a^	0.45 ^a^	0.01	<0.001
F/G	4.91	4.66	5.41	0.17	0.163

^1^ F/G = Feed-to-gain ratio; ADG = average daily gain. ^2^ CON = Infusion with saline only; AMG = infusion with saline and supplementation with *Allium mongolicum* Regel; RTG = infusion with rumen fluid from donor sheep fed with *Allium mongolicum* Regel. ^a^ or ^b^ Within a row, means without a common superscript differ at *p* < 0.05.

**Table 4:** Effects of supplementation with *Allium mongolicum* Regel or rumen fluid transplantation on serum immune of lambs.

Item ^1^	Treatments ^2^			SEM	*p*-Value
	CON	AMG	RTG		
**Day 0**					
IFN-γ (pg/mL)	54.43	56.69	57.03	1.52	0.465
IL-1β (pg/mL)	138.66	122.33	122.63	7.21	0.259
IL-2 (pg/mL)	148.29	138.98	140.47	4.67	0.384
IL-6 (pg/mL)	180.71	164.01	162.76	7.59	0.249
IL-10 (pg/mL)	223.28	242.04	247.44	7.41	0.132
IgA (mg/dL)	30.21	31.70	30.86	0.55	0.238
IgM (mg/dL)	13.41	13.74	13.86	0.20	0.317
IgG (mg/dL)	1319.11	1374.69	1365.56	18.37	0.217
TNF-α (IU/mL)	1093.81	1070.81	1086.98	23.73	0.788
**Day 30**					
IFN-γ (pg/mL)	57.73	60.77	59.73	1.02	0.180
IL-1β (pg/mL)	127.83	111.81	112.33	4.84	0.096
IL-2 (pg/mL)	135.34	129.88	130.02	2.42	0.268
IL-6 (pg/mL)	165.51	155.91	157.84	4.08	0.286
IL-10 (pg/mL)	247.55 ^b^	260.92 ^a^	263.66 ^a^	3.62	0.041
IgA (mg/dL)	29.53 ^b^	34.09 ^a^	33.20 ^a^	0.49	<0.001
IgM (mg/dL)	13.47 ^b^	14.73 ^a^	15.11 ^a^	0.24	<0.001
IgG (mg/dL)	1358.16 ^b^	1423.79 ^a^	1451.72 ^a^	22.97	0.027
TNF-α (IU/mL)	1059.23	1048.57	1044.43	7.59	0.418
**Day 60**					
IFN-γ (pg/mL)	57.46	55.33	56.94	0.83	0.247
IL-1β (pg/mL)	130.10	130.86	130.44	0.98	0.863
IL-2 (pg/mL)	136.98	137.23	131.98	1.57	0.096
IL-6 (pg/mL)	180.42	179.20	178.73	1.71	0.779
IL-10 (pg/mL)	241.94	245.78	246.44	1.55	0.165
IgA (mg/dL)	30.23 ^b^	33.62 ^a^	33.73 ^a^	0.46	<0.001
IgM (mg/dL)	13.49 ^b^	14.86 ^a^	14.65 ^a^	0.25	0.003
IgG (mg/dL)	1334.35 ^b^	1503.18 ^a^	1501.36 ^a^	21.31	<0.001
TNF-α (IU/mL)	1123.64 ^a^	954.89 ^b^	934.18 ^b^	13.82	<0.001

^1^ IFN = Interferon-γ; IL-1β = interleukin-1β; IL-2 = interleukin-2; IL-6 = interleukin-6; IL-10 = interleukin-10; IgA = immunoglobulin A; IgM = immunoglobulin M; IgG = immunoglobulin G; TNF-α = tumor necrosis factor α. ^2^ CON = Infusion with saline only; AMG = infusion with saline and supplementation with *Allium mongolicum* Regel; RTG = infusion with rumen fluid from donor sheep fed *Allium mongolicum* Regel. ^a^ or ^b^ Within a row, means without a common superscript differ at *p* < 0.05.

**Table 5:** Effects of supplementation with *Allium mongolicum* Regel or rumen fluid transplantation on serum antioxidants in lambs.

Item ^1^	Treatments ^2^			SEM	*p*-Value
	CON	AMG	RTG		
**Day 0**					
T-AOC (U/mL)	9.81	10.35	10.27	0.35	0.521
T-SOD (pg/mL)	279.41	295.18	295.14	7.21	0.276
CAT (ng/L)	261.10	289.42	287.82	8.96	0.121
GSH-Px (pmol/mL)	73.35	80.55	79.49	3.00	0.258
MDA (nmol/mL)	13.74	12.51	13.11	0.44	0.220
**Day 30**					
T-AOC (U/mL)	10.39	10.65	10.87	0.18	0.254
T-SOD (pg/mL)	313.53	320.80	318.66	5.49	0.691
CAT (ng/L)	286.86	302.34	302.38	5.46	0.146
GSH-Px (pmol/mL)	81.99	85.71	85.49	1.33	0.165
MDA (nmol/mL)	13.78 ^a^	11.23 ^b^	10.90 ^b^	0.53	<0.001
**Day 60**					
T-AOC (U/mL)	9.90 ^b^	10.70 ^a^	11.04 ^a^	0.13	<0.001
T-SOD (pg/mL)	290.68	292.44	294.35	4.08	0.819
CAT (ng/L)	265.35 ^b^	282.10 ^a^	285.02 ^a^	2.72	<0.001
GSH-Px (pmol/mL)	76.39	77.89	79.91	1.02	0.082
MDA (nmol/mL)	14.21 ^a^	11.18 ^b^	10.69 ^b^	0.24	<0.001

^1^ T-AOC = Total antioxidant capacity; T-SOD = total superoxide dismutase; CAT = catalase; GSH-Px = glutathione peroxidase; MDA = malondialdehyde. ^2^ CON = Infusion with saline only; AMG = infusion with saline and supplementation with *Allium mongolicum* Regel; RTG = infusion with rumen fluid from donor sheep fed *Allium mongolicum* Regel. ^a^ or ^b^ Within a row, means without a common superscript differ at *p* < 0.05.

**Table 6:** Effects of supplementation with *Allium mongolicum* Regel or rumen fluid transplantation on serum biochemical indicators in lambs.

Item ^1^	Treatments ^2^			SEM	*p*-Value
	CON	AMG	RTG		
**Day 0**					
GLU (mmol/L)	3.83	3.60	3.86	0.24	0.712
TP (g/L)	53.11	53.06	50.85	1.35	0.449
ALB (g/L)	26.05	26.36	25.03	1.11	0.691
ALT (U/L)	12.49	12.59	11.16	1.55	0.784
AST (U/L)	100.63	104.96	110.53	5.54	0.490
ALP (U/L)	318.51	354.08	316.40	29.89	0.627
HDL-C (mmol/L)	1.38	1.25	1.05	0.11	0.128
LDL-C (mmol/L)	0.64	0.43	0.46	0.09	0.215
TG (mmol/L)	0.48	0.38	0.36	0.04	0.162
LDH (U/L)	392.69	405.46	418.13	15.12	0.525
BUN (mmol/L)	4.80	5.69	5.97	0.49	0.246
**Day 30**					
GLU (mmol/L)	3.83	3.60	3.89	0.18	0.482
TP (g/L)	45.68	44.94	46.62	2.67	0.907
ALB (g/L)	24.19	24.31	25.84	1.14	0.551
ALT (U/L)	10.25	10.25	10.83	1.22	0.928
AST (U/L)	91.56	88.16	86.89	5.92	0.851
ALP (U/L)	396.43	401.48	408.87	27.81	0.951
HDL-C (mmol/L)	1.15	1.02	1.18	0.08	0.337
LDL-C (mmol/L)	0.38	0.39	0.37	0.03	0.909
TG (mmol/L)	0.32	0.27	0.28	0.02	0.290
LDH (U/L)	466.21	452.09	467.83	29.05	0.916
BUN (mmol/L)	4.45	5.49	4.46	0.41	0.160
**Day 60**					
GLU (mmol/L)	4.08	4.89	3.99	0.34	0.152
TP (g/L)	58.32	69.93	58.16	3.90	0.528
ALB (g/L)	29.31	30.79	28.95	1.59	0.700
ALT (U/L)	9.96	10.90	9.16	1.69	0.776
AST (U/L)	93.68	92.80	80.32	6.52	0.337
ALP (U/L)	443.78	400.97	417.82	41.21	0.769
HDL-C (mmol/L)	1.16 ^b^	1.50 ^a^	0.99 ^b^	0.11	0.013
LDL-C (mmol/L)	0.68 ^a^	0.47 ^b^	0.33 ^b^	0.05	0.010
TG (mmol/L)	0.37	0.37	0.36	0.02	0.900
LDH (U/L)	525.02	589.69	571.21	44.42	0.597
BUN (mmol/L)	6.41 ^a^	4.73 ^b^	3.91 ^b^	0.33	<0.001

^1^ GLU = Glucose; TP = total protein; ALB = albumin; ALT = alanine aminotransferase; AST = aspartate aminotransferase; ALP = alkaline phosphatase; HDL-C = high-density lipoprotein; LDL-C = low-density lipoprotein; TG = triglyceride; LDH = lactate dehydrogenase; BUN = blood urea nitrogen. ^2^ CON = Infusion with saline only; AMG = infusion with saline and supplementation with *Allium mongolicum* Regel; RTG = infusion with rumen fluid from donor sheep fed *Allium mongolicum* Regel. ^a^ or ^b^ Within a row, means without a common superscript differ at *p* < 0.05.

## Data Availability

Dataset available on request from the authors.
